# Desorption Kinetics Evaluation for the Development of Validated Desorption Electrospray Ionization-Mass Spectrometric Assays for Drug Quantification in Tissue Sections

**DOI:** 10.3390/ijms24108469

**Published:** 2023-05-09

**Authors:** Margaux Fresnais, Siwen Liang, Deniz Seven, Nevena Prodanovic, Julia Sundheimer, Walter E. Haefeli, Jürgen Burhenne, Rémi Longuespée

**Affiliations:** 1 Department of Clinical Pharmacology and Pharmacoepidemiology, Heidelberg University Hospital, Im Neuenheimer Feld 410, 69120 Heidelberg, Germany; 2 Hopp Children’s Cancer Center Heidelberg (KiTZ), Im Neuenheimer Feld 430, 69120 Heidelberg, Germany; 3 Division of Pediatric Neurooncology, German Cancer Research Center (DKFZ), German Cancer Consortium (DKTK), Im Neuenheimer Feld 280, 69120 Heidelberg, Germany; 4 Faculty of Biosciences, Heidelberg University, Im Neuenheimer Feld 234, 69120 Heidelberg, Germany

**Keywords:** desorption electrospray ionization, mass spectrometry, drug quantification

## Abstract

The development of desorption/ionization (DI) mass spectrometric (MS) assays for drug quantification in tissue sections and their validation according to regulatory guidelines would enable their universalization for applications in (clinical) pharmacology. Recently, new enhancements in desorption electrospray ionization (DESI) have highlighted the reliability of this ion source for the development of targeted quantification methods that meet requirements for method validation. However, it is necessary to consider subtle parameters leading to the success of such method developments, such as the morphology of desorption spots, the analytical time, and sample surface, to cite but a few. Here, we provide additional experimental data highlighting an additional important parameter, based on the unique advantage of DESI-MS on continuous extraction during analysis. We demonstrate that considering desorption kinetics during DESI analyses would largely help (i) reducing analytical time during profiling analyses, (ii) verifying solvent-based drug extraction using the selected sample preparation method for profiling and imaging modes, and (iii) predicting the feasibility of imaging assays using samples in a given expected concentration range of the targeted drug. These observations will likely serve as precious guidance for the development of validated DESI-profiling and imaging methods in the future.

## 1. Introduction

On-surface analyses are particularly useful for the quantification of drugs at their sites of action [1] in tissue sections, in a preserved histological context, and with a limited intervention for sample preparation. For instance, in brain cancer research, direct quantification in tissue section allows to determine if the drug crosses the blood brain barrier and if it reaches the intended tumor site [2]. Profiling and imaging mass spectrometry (MS) modes are available to the users in order to define the best balance between sensitivity and spatial resolution. Different MS modalities applied on tissue surfaces allow to access the most adequately different compound classes. While drug quantification is possible using secondary ion MS [3,4,5,6], laser ablation-nductively coupled plasma MS (for metal-containing drugs) [3,5,7,8,9], and a large panel of additional sources, matrix-assisted laser desorption/ionization (MALDI) [5,10,11] and desorption electrospray ionization (DESI) [5,12] are the most universal MS sources used for on-surface drug quantification. Using adapted considerations for method validation, on-surface MS methods are in the process of being considered as reliable members of the panel of methods for drug quantification in (pre)clinical studies [13]. Method validation has already been largely approached for MALDI imaging [14] and profiling approaches [15], and DESI is in the process of being considered as method of choice for method development [16].

DESI-MS has long suffered from the lack of robust hardware for the development of drug quantification assays in tissue sections [17,18], but recent enhancements have been proven to be highly valuable for the development of methods that follow the requirements of regulatory guidelines [16,19]. The most recent advances were performed in profiling mode, in which analyses are performed in selected locations with known histology. Profiling mode is opposed to the imaging mode, in which the whole surface of the sample is analyzed to further correlate ion images with histological information. Profiling mode permits to evaluate initial best-case scenarios for method development of desorption ionization (DI)-MS methods. Indeed, with profiling mode it is possible to use larger volumes of solvents for sample preparation to more efficiently extract drugs from tissues, and extend analysis times to cover a larger sample surface, as compared to imaging mode. This allows for the accumulation of ions from the compounds of interest for spectral collection in order to reach higher sensitivity in developed assays while respecting histological information. Using the most universal DI-MS approach, MALDI, extraction of compounds only takes place during sample preparation. DESI-MS differs in that the extraction of the targeted compounds partly relies on the desorption process in the source and not only on sample preparation. In principle, this allows for the generation of desorption kinetic data during the analysis, and it helps control when the analyte extraction is the most efficient for the development of quantification assays. In the present manuscript, we report desorption kinetics of DESI-MS profiling assays for the quantification of the ERK 1/2 inhibitor ulixertinib (ULN), recently demonstrated as a candidate for the treatment of pediatric low-grade gliomas [20].

This study had two primary objectives: (i) evaluate desorption kinetics to optimize DESI-MS assays for drug quantification, and (ii) assess drug extraction induced by the sample preparation and the desorption process in order to control the proper mixture of extracted drugs with the internal standard (IS) during the analysis of dosed tissues. First, desorption kinetics informed on the minimal signal accumulation necessary for the creation of calibration curves. Second, we also initiated the creation of mimetic dosed tissues by immerging control mouse brains in ULN solutions in order to evaluate the impact of solvent-based extraction during sample preparation and the DESI process. This evaluation is particularly useful in order to control the preparation of dosed tissues spiked with IS or for the preparation of calibration standards (CALs) and quality control (QC) samples. Drugs from dosed tissues are extracted from the tissue section, while IS, or the reference compounds used for CALs and QCs, are deposited at its surface. A suboptimal solvent-based extraction of drugs from the tissue would thus lead to an underestimation of their concentration [16,21].

Finally, this study of desorption kinetics assesses the feasibility of DESI imaging assays using pharmacologically-active concentrations of drugs, and it aims to give precious hints for the future development and optimization of DESI-MS profiling and imaging methods for drug quantification.

## 2. Results

**Figure 1:**
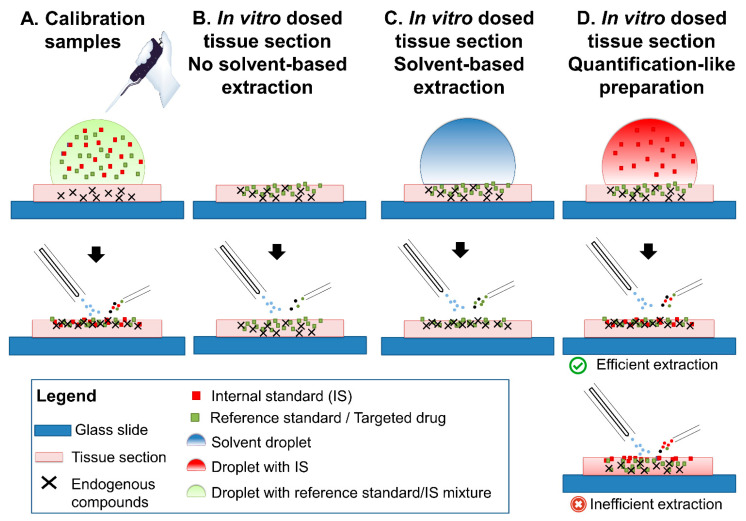
Different samples and sample preparation used for the study and hypotheses for extraction (**A**). Calibration samples, (**B**). In vitro-dosed tissue sections without extraction, (**C**). In vitro-dosed tissue sections with extraction, (**D**). In vitro-dosed tissue sections as prepared for quantification assays.

Different types of tissue material and sample preparations were performed to achieve the goals described in the introduction. Figure 1 summarizes all the types of preparations used. Only profiling approaches were used.

### 2.1. Tissue Morphology Kinetics

The first point of information gathered was the level of tissue decay induced by the desorption process using an eluent spray. In the case of complete desorption of the tissue thickness until its disappearance, it could be assumed that targeted drug and IS would have been ionized throughout the whole thickness of the tissue. In this case, a suboptimal drug extraction at the surface of the tissue, and the subsequent heterogenous mixture of the drug and its IS throughout the tissue thickness (Figure 1D, lower inset “Inefficient extraction”), might not impact responses for drug quantification.

**Figure 2:**
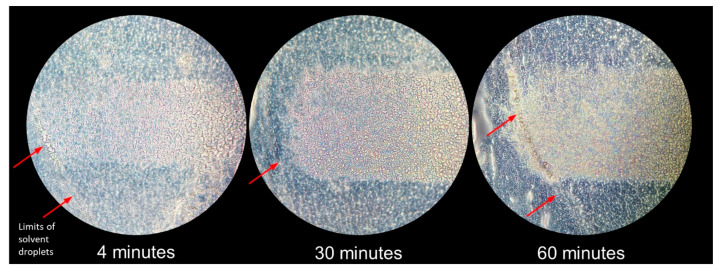
Magnified view of tissue section areas analyzed by desorption electrospray ionization for 4 min, 30 min, and 60 min. Limits of solvent droplets used for the spotting of calibration and internal standards are indicated with red arrows.

However, the magnification on calibration samples (Figure 1A) indicated that tissue material was still remaining after 4 min of analysis (Figure 2). A slurry was formed in the place of the desorption area, even after longer desorption times (4, 30, 60 min, Figure 2), which was likely due to the extended exposure to the solvent mixture. The incomplete tissue decay confirmed the necessity to design strategies to follow the desorption kinetics of the drug and its IS.

### 2.2. Desorption Kinetics

**Figure 3:**
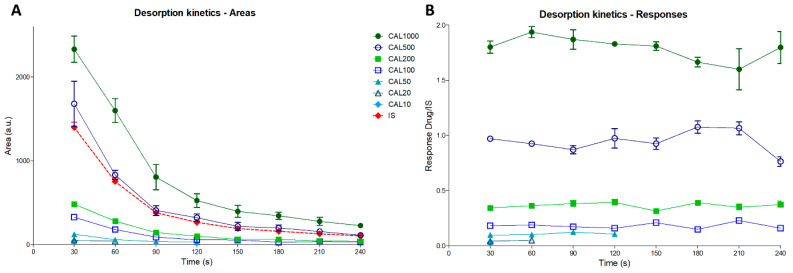
Desorption kinetics curves of desorption electrospray ionization (DESI) analyses of blank mouse brain sections spiked with increasing concentrations of ulixertinib (ULN), based on mean absolute areas of ULN in each concentration level and for each successive time window, as well as mean areas of internal standard ULN-d6 from all samples at each successive time window (**A**), and based on mean ULN responses (ratios of ULN area over ULN-d6 area) in each concentration level and for each time window (**B**). IS, internal standard; CAL, calibration sample.

A kinetic analysis of a calibration batch (i.e., samples prepared as in Figure 1A), formerly published [16], was performed. In calibration samples, a mix of drug and IS were deposited above the tissue section. Kinetic analyses of calibration batches can therefore be considered as templates of homogenously mixed drugs and IS in tissue samples. The results indicated a decrease in drug and IS signals over time (Figure 3A) that can be explained by: (i) the disappearance of the drug during its extraction by the DESI process (i.e., as deeper tissue layers were reached) and (ii) the lower desorption efficiency due to the evolution of the morphology of the tissue material during the desorption analysis. Normalized response (drug signal over IS signal ratio) kinetics at higher concentrations were consistent with a proper mixture of drugs and IS during the analysis (Figure 3B), with constant responses reported over time. An heterogenous mixture of drug and IS during the analysis would, for instance, lead to a response increase if IS dropped at the tissue surface was more accessible, leading to an over-normalization of the signal at the beginning of the analysis [16]. At lower concentrations, a response decrease was observed, corresponding to the complete extinction of the signal from the drug after some minutes of analysis.

### 2.3. Optimal Analysis Time Determination

**Figure 4:**
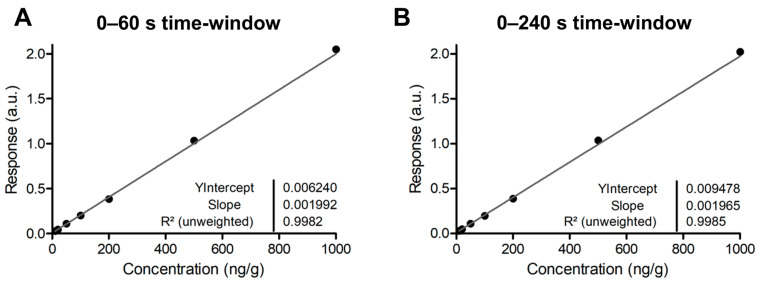
Calibration curves computed using responses from cumulated ion area in extracted ion mobilograms specific to the targeted drug and related internal standard over a 0 to 60 s time window (**A**) and over a 0 to 240 s time window (**B**). Both curves are comparable, thus showing that a 60 s analysis time would be enough for acquisition.

From desorption kinetic data, it is possible to determine the optimal time for profiling analysis by partitioning the analytical batch, for which 4 min acquisitions were performed, in different sub-batches of increasing times (Table 1, “Time window”). The results indicated that a reliable calibration curve including the targeted LLOQ (i.e., 10 ng/g) could be obtained already from the first 60 s window instead of the initial 4 min set for the calibration batch (Table 1, Figure 4). This presents a major advantage for time optimization of DESI profiling assays for drug quantification. This also means that, although the signal rapidly decreased during the desorption process, an accumulated signal with a minimum of 60 s is necessary for the acceptance of the calibration curves. In the present setting of oscillated motion for profiling, each passage over the 2 mm length takes 20 s. Three passages over the same tissue area are therefore necessary to extract enough ion signal for the creation of calibration curves.

### 2.4. Evaluation of Extraction Processes from Dosed Tissues

In order to evaluate whether extraction processes would be appropriate in dosed tissue, we evaluated whether desorption kinetics from dosed tissue follows these from calibration samples.

#### 2.4.1. Differential Extraction Effects from Droplet Deposition and DESI Solvent Spray

**Figure 5:**
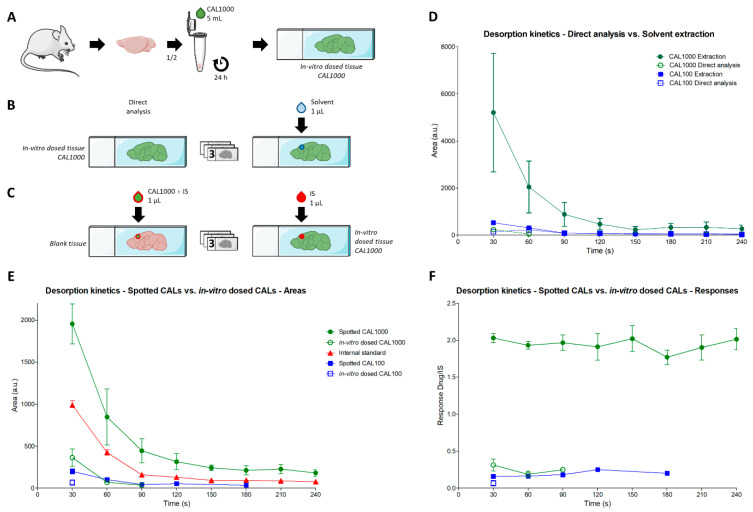
Workflow to produce in vitro dosed mouse brain tissues using calibration standard solutions at 1000 ng/mL (CAL1000) and 100 ng/mL (CAL100) (**A**). Comparison of sample preparation of in vitro dosed tissue at CAL1000 and CAL100 level in triplicates for direct analysis versus for analysis after analyte extraction by spotting 1 µL of blank solvent to mimic conditions used for real samples (**B**). Evaluation of the efficiency of the analyte extraction from triplicate tissue sections during the sample preparation by comparing the ulixertinib (ULN) signal with the associated response obtained from the blank tissue section spiked with 1 µL of CAL1000/internal standard (IS) mixed solution and with the in-vitro dosed tissue section at CAL1000 level spiked with 1 µL of IS solution (**C**). Desorption kinetic curve based on ULN peak area corresponding to experiment presented in B (**D**). Desorption kinetic curves corresponding to experiment presented in C based either on ULN peak area (**E**) or on ULN normalized response (**F**).

The first goal was to determine the effect of the extraction induced by the sample preparation and by the DESI process in dosed tissues. We hypothesized that solvent-based extraction draws the drug to the surface of the tissue for further higher accessibility for desorption (Figure 1B,C). In order to verify this hypothesis, we created in vitro dosed tissues following procedures described before [22] (Figure 5A). In vitro dosed tissues were previously suggested by our group as possible QC tissue models for the development and validation of DI-MS methods for drug quantification in tissue sections [13]. One of the most important parameters to be controlled for drug quantification method validation using in vitro dosed tissues is the extraction recovery. Evaluation of recovery would permit to verify that drug quantities are not underestimated due to inefficient solvent-based extraction processes during sample preparation [13]. In vitro dosed tissues would represent a valuable alternative to tissue homogenates spiked with drugs [23,24]. In tissue homogenates, microheterogeneities in tissue sections are not preserved, and specific biological matrix effects induced by different molecular contents and surface asperities of regions of interest can thus be overlooked [13]. On the contrary, controlled in vitro dosed tissues would still integrate these heterogeneities [13]. For the creation of the in vitro dosed tissue, we immersed the mouse brain hemisphere in 5 mL of ULN solution at 1000 and 100 ng/mL (CAL1000 and CAL100, respectively). Since the volume of a mouse brain is 0.5 mL, one hemisphere represents only 4.5% of the total volume (i.e., ULN solution and brain hemisphere). We assumed that, after 24 h, an equilibrium between the solution and the tissue would take place and the concentration of the drug in the tissue would reach 1000 ng/g or 100 ng/g when immersed in a 1000 ng/mL or a 100 ng/mL solution, respectively (i.e., the equivalent of CAL1000 and CAL100 tissues).

To test the extraction occurring during the sample preparation process (droplet deposition), analyses from tissue sections were either performed without any sample preparation (Figure 1B), or after depositing only solvent mixture (Figure 1C and Figure 5B). The results indicated that significantly higher signals could be obtained when a solvent extraction was performed at the surface of the tissue, as compared to no extraction (Figure 5D). This confirmed that solvent extraction is necessary to bring drugs to the surface of the dosed tissue sections. Since solid–liquid extraction is more efficient using higher volumes of liquid, higher volumes of deposits provide higher extraction yields.

Although minor, it was confirmed that the DESI process induces an extraction. However, the results also indicated that the same decrease in the kinetics of intensity could be observed during analyses from raw and extracted tissue sections (Figure 5D). Since the same amount of drug is present in both samples, a continued extraction should be observed in non-extracted samples if desorption was kinetically the same during the entire time course of the analysis. Drug disappearance from the tissue section can therefore not explain alone the intensity decrease. This signal decrease thus appears to come from the tissue morphology changes during the analysis. The slurry that is progressively formed in the tissue section seems to hamper the desorption process.

#### 2.4.2. Evaluation of Response Kinetics in In Vitro-Dosed Tissues

At this step, it can be deduced that although the intensity decrease is due to morphological evolution of the tissue during the DESI analysis, the solvent extraction brings drugs from dosed tissues at the surface of the sections. However, it remains unknown if drugs extracted from sections of dosed tissue would be as accessible to desorption similarly to ISs that are dropped onto the tissue sections. In order to evaluate this, two batches of analyses were performed (Figure 5C). As described above, results from CAL samples can be considered as the template scenario when drugs are efficiently extracted and thus optimally mixed with their deposited IS. One series of blank tissue sections was then prepared as CAL samples (Figure 1A), i.e., dropped with the drug at known concentrations and the IS. For the “real case” in vitro dosed samples, the drug was extracted from the tissue sections and IS dropped on top (Figure 1D). For the CAL samples, consistent results were obtained as compared to former ones from the evaluation of the batch for calibration (Figure 5E,F). The in vitro dosed samples indicated similar results as with low-concentration CAL samples, with significantly lower signals for the drugs (Figure 5E) and stable responses over time (Figure 5F). Two assumptions could explain this observation: (i) the drug concentration in the dosed tissue is much lower than expected, or (ii) only a portion of the drug is extracted from the dosed tissue. However, the stable response over time might suggest a proper mixture with the IS and, therefore, a proper extraction of the drug at the surface of the tissue. This informs about future needs for the development of QCs and their use to control the extraction of drugs from sections: (i) the development of in vitro dosed tissues for QCs must be assisted by LC-MS/MS to control the concentration of drugs for each studied mouse brain, possibly with the help of macro/microdissection to measure differential concentrations in regions of interest (ROIs), (ii) once concentration is defined in known ROIs, extraction recovery should be controlled during the development of the methods.

### 2.5. Feasibility and Parameter Predictions for DESI-Imaging

**Figure 6:**
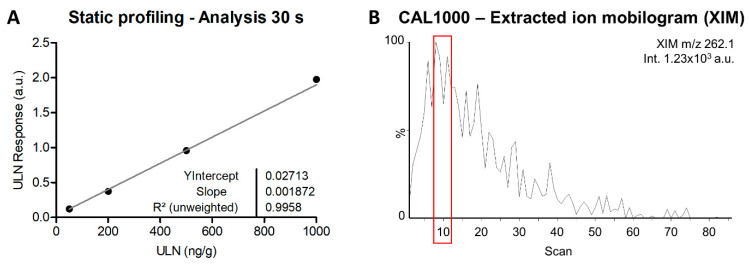
Calibration curve over four accepted concentration levels (out of the minimum of six levels requested by regulatory guidelines) using static profiling acquisition mode over 30 s (**A**). Detailed quantification data are summarized in Table 2. Extracted ion mobilogram (XIM) specific to ulixertinib *m/z* 262.1 peak showing maximum of intensities around 10 scans (i.e., about 5 s of analysis). The red box indicates when the highest signal for ulixertinib is reached (**B**).

Imaging mode requires significantly smaller volumes of solutions to deposit reference compounds and IS and to allow extraction without generating inter-pixel contamination (also called molecular delocalization). It also implies that we should analyze significantly smaller volumes of samples from pixels that will be further reconstituted in chemical maps. In addition, a constant motion has to be applied to record signals from each pixel. For these reasons, LLOQs in DI-imaging assays are usually much higher as compared to profiling modes [25]. In order to pre-evaluate the feasibility and critical parameters for DESI imaging assays respecting regulatory guidelines given by the International Council for Harmonisation (ICH) [19], static profiling [17] batches can be performed in order to estimate reachable LLOQ, precisions, and accuracies (Table 2 and Figure 6A), as well as strategies to obtain the maximal signal from single pixels (Figure 6B). The static profiling assays indicated that, when using these experimental settings, the calibration batch would not be accepted, following the ICH guidelines, with an LLOQ of 50 ng/g, which is 5× superior to the one obtained with oscillated profiling and precision and accuracy values. This could also limit the reliability of the assay, even at higher concentration levels (e.g., CAL100 and CAL200). Desorption kinetics evaluation also permits to estimate the necessary time for sample solvation for the formation of secondary droplets [26], as well as the optimal time for signal acquisition. The maximal intensity was obtained after about 10 scans of 0.5 s (i.e., 5 s) and a relatively intense signal was recorded 10 s after (Figure 6B). This indicated the necessity to set imaging assays with slow-motion parameters. Further evaluations could also be performed with oscillated profiling using the selected motion parameters and evaluating the obtained signals within the time window corresponding to the time of analysis of one pixel at the chosen spatial resolution. Information from static profiling, together with the smaller volumes used for standard deposition suggest that LLOQ higher than 50 ng/g could still be expected for DESI imaging assays, and that accurate and precise results as defined by the ICH guidelines could still be challenging to reach.

## 3. Discussion

In the present manuscript, we illustrated the possible advantages of evaluating the kinetic aspects of DESI analyses for the development of profiling and imaging methods. Our initial observations after different desorption times indicated that the whole thickness of a tissue section was not completely “consumed” during analyses. This observation suggested that evaluating drug extraction from dosed tissue sections by sample preparation would be relevant in order to ensure that the drug amounts analyzed from the tissue surface are representative of their content in the whole tissue thickness. Desorption kinetics from calibration samples (i.e., the drug standard and its IS are dropped on top of the tissue section) indicated that a dramatic signal decrease takes place, likely due to the change of tissue morphology during the desorption process. However, constant responses were observed, indicative of a proper mixture between the standard and the IS. The development of mimetic in vitro dosed tissues permitted to understand the fundamental aspects of drug extraction that are enhanced during the sample preparation and desorption process. First, analytical comparisons between sections of in vitro dosed tissues subjected, or not, to the deposition of the solvent droplet indicated that the sample preparation actually brings the drug to the surface of sections in dosed tissues. Second, comparison of calibration samples to in vitro dosed tissues spiked with the IS preliminary suggested a constant response, possibly indicative of a proper mixture of the drug with the IS, as with the calibration samples. However, further developments of in vitro dosed tissues will be necessary to provide final conclusions.

Dosed mimetic tissues would represent an important asset in evaluating extraction efficiency from tissues, and it could, therefore, support method validation, for which extraction recovery is a necessary parameter for the evaluation of different subregions of interest of tissue sections. The advantage of in vitro dosed tissues, as compared to tissue homogenates [23,24], are the following: (i) tissue homogeneization is itself a form a lysis and may influence the interaction of the drug with the tissue, (ii) the structure of tissue homogenates are different from a “native” tissue, and differences in morphological microheterogeneities might greatly influence the desorption, (iii) the tissue homogenates reflect only a global concentration and not concentrations in subregions of tissues, and (iv) after proper method development, in vitro dosed tissues should, in principle, mimic the equilibrium of drug diffusion existing within a body. As stated above, further developments would be necessary for the development of in vitro dosed tissues. In the present study, it appears more likely that the diffusion of the drug in 24 h at 4 °C was limited, thus explaining the low signals obtained for ULN. Further development would then be necessary, e.g., testing different parameters for in vitro dosing (temperature, time, etc.) and systematically verifying (i) that the diffusion of the drug is homogenous trough the tissue and (ii) the concentration of drugs in each ROI. The homogeneity of diffusion (i) could be assessed by DESI-MS in imaging mode [22], while the concentration of drugs in different tissue ROIs (ii) could be assessed by the combination of laser microdissection (LMD) and LC-MS [27,28,29,30,31]. In the present study, even with unknown concentrations in “preliminary” in vitro dosed tissues, this sample material allowed for the understanding of important fundamental desorption processes that will likely influence further development of DESI-MS assays for drug quantification.

Moreover, evaluating desorption kinetics allowed for the determination of the minimum analytical time to produce calibration curves that would meet requirements from regulatory guidelines. These parameters allow for a considerable time optimization of profiling assays, with 4× shorter analyses. While profiling assays give the “best case scenario” for drug quantification, validated DESI imaging will be more challenging. Since MS imaging analyses are performed from significantly lower sample volumes, within shorter times, and using lower solvent volumes for drug extraction, the evaluation of desorption kinetics also allows to predict their feasibility.

## 4. Material and Methods

### 4.1. Chemicals

MS-grade H_2_O, organic solvents, and formic acid (FA) were purchased from Biosolve Chimie SARL (Dieuze, France). Solutions of ULN and its deuterated IS, ULN-d_6_, were provided by BioMed Valley Discoveries (Kansas City, MO, USA) with purities of 99% and 98.26%, respectively [16].

### 4.2. Solutions Preparation

Calibrant solutions preparation and solutions deposition on tissue sections were performed as detailed before [16]. Briefly, sub-stock solutions of ULN and its IS, ULN-d_6_, were prepared at 20 µg/mL in MeOH/H_2_O 1:1 (*v*/*v*). From the sub-stock solution, calibration standard (CAL) solutions of ULN on seven non-zero levels were prepared with serial dilution in the solvent mixture MeOH/H_2_O 1:1 (*v*/*v*). For each CAL level, the final solution to deposit on tissue (dilution mix) was prepared by mixing the corresponding ULN CAL solution and the IS sub-stock solution with solvent (CAL/IS/solvent mixture 1.76:1.76:46.4).

### 4.3. Animal Dissection

For all samples, untreated female NSG mice, aged 10 weeks and weighing 25 g, were euthanized, and their brains were dissected and snap-frozen in liquid nitrogen. All killings for organ removal were performed according to German Laws for Animal Protection and approved by the responsible animal welfare officer (internal reference number DKFZ374).

Frozen brains were divided longitudinally into two hemispheres. Blank tissues were directly snap frozen and stored at −80 °C, and in vitro dosed tissues were prepared as described below (Section 4.4).

### 4.4. In Vitro Dosed Tissue Preparation

For in vitro dosed tissues, the hemispheres were directly separated after dissection and immersed in 5 mL of ULN solutions at different concentrations corresponding to CAL100 and CAL1000, all dissolved in PBS. The brains were left under gentle mixing at 4 °C for 24 h. Thereafter, the hemispheres were rinsed twice rapidly in PBS and snap-frozen in liquid nitrogen before storage at −80 °C.

### 4.5. Tissue Sectioning

From whole hemispheres, 10 µm-thick serial sections were made using a Leica CM 1950 UV cryostat (Leica Biosystems GmbH, Wetzlar, Germany) and stored at −80 °C before sample preparation.

### 4.6. Solution Deposition on Tissues

In order to ensure reliable analytical comparisons, depositions were performed on serial tissues section for each analytical batch. Depositions were only performed on cortex areas large enough to avoid any overlap with adjacent regions.

For each dilution mix solution of ULN CALs (Figure 1A), as well as for solvent (Figure 1C) or IS (Figure 1D) deposition on in vitro-dosed tissues, 1 µL of solution was deposited onto a tissue section, and three up-and-down pipetting motions were performed in order to extract endogenous compounds from the tissue in the deposition area. For CAL samples, the final tissue concentrations ranged from 10 ng/g (i.e., CAL10) to 1000 ng/g (i.e., CAL1000) [16]. For evaluation of tissue morphology kinetics, CAL10 samples were used arbitrarily, since the concentration was not a relevant parameter for the evaluation.

### 4.7. Mass Spectrometric Analyses

The analyses were performed with a SYNAPT G2-Si instrument (Waters Corporation, Wilmslow, UK) consisting of an orthogonal acceleration (oa)-quadrupole (Q)-ion mobility (IM)-time-of-flight (TOF) mass spectrometer equipped with an enhanced DESI source formerly described [16] and controlled using MassLynx v.4.1 (Waters Corporation, Wilmslow, UK). The source was used in profiling mode, with an oscillation length of 2 mm and speed of 100 µm/s. The solvent (MeOH/H_2_O 95:5) flow was set to 3 µL/min, as described before [16].

The instrument was used in “resolution” mode (“W” mode) and instrument calibration was performed in TOF-MS/MS mode using leucine enkephalin deposited on Aquarray slides (Aquarray GmbH, Eggenstein-Leopoldshafen, Germany), as described before [16]. For analytical development, the instrument was used in the IM mode. Previously described IM-MS/MS parameters were used, consisting of the quadrupole selection of a specific parent ion followed by collision-induced dissociation (CID) at 32 V and subsequent IM separation of the fragments before MS detection (Method 4 [15]). ULN was analyzed by focusing the quadrupole on mass-over-charge (*m*/*z*) 433 and monitoring the ion mobility peak relying on the ULN fragment at *m*/*z* 262 [16].

### 4.8. Data Processing

Mobilograms and MS spectra were extracted from MassLynx v.4.1 (Waters Corporation, Wilmslow, UK), and desorption kinetic curves were computed using Prism software v.5.01 (GraphPad, La Jolla, CA, USA). Recommendations were followed to report IM-MS measurements [32]. IM was used as a separation method for post-acquisition signal filtering, which consists of extracting the ion mobility time range specific to the compounds of interest from the complete ion mobility time range for further data processing for quantification [15]. Since IM is used here as a separation method and not for structural analyses, the drift times (DT) are reported as IM data [32]. For desorption kinetics data extraction, two-dimensional mobility maps (*m*/*z* vs. DT) were obtained using Driftscope TM v.2.9 (Waters Corporation, Wilmslow, UK) by cumulating signals of successive 30 s windows for each analysis. The previously described MobA method was used for data extraction [15]: the mobilograms of the compounds of interest were first extracted from the regions of the mass spectra specific to each in order to obtain the specific extracted ion mobilograms (XIM). The obtained XIM were then automatically integrated to retrieve the peak areas using MassLynx software v.4.1 (Waters Corporation, Wilmslow, UK) [15]. Each desorption kinetics curve was created by plotting the signal of each of the successive 30 s windows from a single analysis. Normalized responses were calculated using the ratio of the ULN mobility peak area to corresponding IS mobility peak area. Data extraction was automated using the Chrotool feature from MassLynx to obtain the different XIMs (i.e., automatic extraction of the specific XIMs from the full mobilogram).

### 4.9. Analytical Quality Parameters

In clinical laboratories, analytical methods are developed and validated following the regulatory guidelines from the ICH [19]. If this is an obligation in clinical studies, these quality principles are also generally followed in preclinical contexts in order to ensure the use of reliable and reproducible quantitative bioanalytical assays. ICH M10 guidelines were designed for chromatography-based assays (e.g., LC-MS/MS), but the different parameters can also be applied to DI-MS methods. The validation of DI-MS methods was approached in detail in a previous work [13], but the main parameters of interest for the present study are reproduced here.

In the present study, quality parameters were set to assess the reliability of specific settings, not to reach a full method validation. Therefore, only a few critical parameters were considered, and related ICH M10 guidelines were strictly followed without adaptations, including the quality parameter calculations:

(i) Calibration curve: There should be a minimum of six accepted non-zero concentration levels, and linearity should be proven over the full calibration range using the same regression model. As a in-study rule, linear regression with 1/x^2^ weighing was always applied and a valid determination factor r^2^ should be >0.985.
(ii) Accuracy: Each calibration sample was accepted only with an accuracy of ±15% bias, except at the lower limit of quantification (LLOQ), where an accuracy of ±20% bias should be achieved.
(iii) Precision: Each calibration level was accepted if the precision between replicates was <15% CV or <20% CV at the LLOQ, and if at least 50% of replicates were accepted.
(iv) Total accepted replicates: These should be at least 75% of measured calibration standard replicates.

## 5. Conclusions

DESI-MS presents the unique advantage of allowing the monitoring of the kinetics of drug analyses in tissues. Here, it is suggested to exploit this advantage for assay optimization, extraction evaluation, and imaging feasibility studies.

## Figures and Tables

**Table 1:** Summary of calibration results obtained from each cumulated time-window over the four minutes of analysis of a calibration batch from CAL10 to CAL1000 (seven non-zero levels in triplicate).

Time Window	Accepted ^1^ Levels	Accepted ^1^ Replicates	LLOQ	Mean Area at LLOQ	Accuracies (% Bias)	Precision (% CV)	R^2^
**0–30 s**	6/7	86%	CAL20	48	−11.8–10.1	0.8–9.8	0.9959
**0–60 s**	7/7	95%	CAL10	51	−12.0–14.0	1.3–18.0 ^2^	0.9982
**0–90 s**	7/7	95%	CAL10	61	−7.8–10.7	0.5–10.0	0.9988
**0–120 s**	7/7	95%	CAL10	68	−9.2–10.9	0.3–8.3	0.9986
**0–150 s**	7/7	95%	CAL10	75	−14.7–10.7	0.5–10.8	0.9982
**0–180 s**	7/7	95%	CAL10	78	−10.9–12.0	0.1–10.1	0.9988
**0–210 s**	7/7	95%	CAL10	80	−9.8–12.2	0.7–10.9	0.9989
**0–240 s**	7/7	95%	CAL10	85	−10.7–12.0	0.7–11.3	0.9985

CV: coefficient of variation; LLOQ: lower limit of quantification. ^1^ Individual replicates are accepted if they meet the ±15% bias criterium for accuracy, or ±20% bias at LLOQ. Concentration levels are accepted if at least 50% of the replicates for that level are accepted. ^2^ The 18% CV precision between replicates was obtained for the LLOQ level, with two accepted replicates out of three.

**Table 2:** Summary of calibration results for ulixertinib (ULN) quantification in mouse brain sections using static profiling acquisition mode over 30 s analysis.

Level	ULN Concentrations (ng/g)			Accuracies (% Bias)			Mean Concentration (ng/g)	Standard Deviation (ng/g)	Precision (% CV)
	1	2	3	1	2	3			
**CAL10**	*25.43* ^1^	*−14.49*	*5.66*	*154.3*	*−244.9*	*−43.4*	5.53	19.96	360.9
**CAL20**	*50.74*	*35.79*	*57.18*	*153.7*	*79.0*	*185.9*	47.91	10.97	22.9
**CAL50**	56.30	*68.26*	45.25	12.6	*36.5*	−9.5	50.77	7.81	15.4
**CAL100**	*138.25*	*101.88*	*75.95*	*38.2*	*1.9*	*−24.1*	105.36	31.30	29.7
**CAL200**	*252.47*	184.45	187.01	*26.2*	−7.8	−6.5	185.73	1.81	1.0
**CAL500**	*593.59*	493.92	499.16	*18.7*	−1.2	−0.2	496.54	3.70	0.7
**CAL1000**	1050.73	927.44	1024.91	5.1	−7.3	2.5	1001.03	65.02	6.5
**Slope**	0.001872			**Intercept**		0.02713	**R^2^**	0.9958	

^1^ *xxx* Strikethrough values were excluded because of accuracies outside the ±15% bias limits or because two out of three value of the same level were already excluded (i.e., full level excluded when more than 50% of values are excluded).

## Data Availability

Data are reported in the main text.
